# Development of Three-Dimensional (3D) Biodegradable Polyglycolic Acid Fiber (PGA) Preforms for Scaffold Applications: Experimental Patterning and Fiber Volume Fraction-Porosity Modeling Study

**DOI:** 10.3390/polym15092083

**Published:** 2023-04-27

**Authors:** Hikmet Kundak, Kadir Bilisik

**Affiliations:** 1 Nano/Micro Fiber Preform Design and Composite Laboratory, Department of Textile Engineering, Faculty of Engineering, Erciyes University, Talas 38039, Kayseri, Turkey; hikmet.kundak1@gmail.com; 2 Nanotechnology Application and Research Centre (ERNAM), Erciyes University, Talas 38039, Kayseri, Turkey

**Keywords:** 3D orthogonal preform, polyglycolic acid (PGA) fiber, fiber volume–porosity fraction, stiffness and strength, scaffolds

## Abstract

Three-dimensional (3D) biodegradable polyglycolic acid fiber (PGA) preforms were developed as temporary scaffolds for three-dimensional tissue regeneration applications. Three-dimensional biodegradable polyglycolic acid fiber (PGA) preforms including various degrees of interlaced structures called 3D plain, semi-interlaced, and orthogonal woven preforms were designed. Analytical relations and finite element model-based software (TexGen) on fiber volume fraction and porosity fraction were proposed to predict scaffolds’ stiffness and strength properties considering micromechanics relations. It was revealed that yarn-to-yarn space, density, and angles of all 3D PGA fiber preforms were heterogeneous and demonstrated direction-dependent features (anisotropy). Total fiber volume fractions (*V_fp_*) and porosity fraction (*V_tpr_*) predicted by analytic and numerical modelling of all 3D scaffolds showed some deviations compared to the measured values. This was because yarn cross-sections in the scaffolds were changed from ideal circular yarn (fiber TOW) geometry to high-order ellipse (lenticular) due to inter-fiber pressure generated under a tensile-based macrostress environment during preform formation. Z-yarn modulus (*E_z-yarn_*) and strength (*σ_z-yarn_*) were probably critical values due to strong stiffness and strength in the through-the-thickness direction where hydrogel modulus and strengths were negligibly small. Morphology of the scaffold showed that PGA fiber sets in the preform were locally distorted, and they appeared as inconsistent and inhomogeneous continuous fiber forms. Additionally, various porosity shapes in the preform based on the virtual model featured complex shapes from nearly trapezoidal beams to partial or concave rectangular beams and ellipsoid rectangular cylinders. It was concluded that 3D polyglycolic acid fiber preforms could be a temporary supportive substrate for 3D tissue regeneration because cells in the scaffold’s thickness can grow via through-the-thickness fiber (z-yarn), including various possible mechanobiology mechanisms.

## 1. Introduction

Medical technologies including tissue engineering, tissue regeneration and cell therapies, organ printing and cell patterning, a range of drug and gene delivery systems, and nanotechnology-based imaging and diagnostic systems are intensively researched by various universities, organizations, and state laboratories [1]. These materials encompass conventional metals, ceramics, and synthetic polymers including contemporary materials such as biopolymers, graphene nanoplatelets (GNPs), carbon nanotubes (CNTs), and quantum dots (QDs) [2,3,4,5,6]. The developments of tissue engineering can create engineered tissues, cells, and organs such as skin (scar tissue) and cartilage [1,7]. It is one of the comprehensive subjects in inter- and multidisciplinary areas. It is defined as applying engineering and life science to develop engineering tissue and understand its structure, including functional principles [8]. Scaffold materials and fabrication technologies play a critical role in tissue engineering. Engineered tissues, particularly for reconstructive surgery and transplantation, exhibited remarkable promise in replacing degraded organs [9]. Three-dimensional tissue formations during temporary scaffold design are crucial, especially chosen materials and fabrication methods [9].

It was reported that progress in tissue engineering shows steadily slow growth in terms of clinical outcomes and commercialization. This partly complex integration comprises various phases, from sourcing cells to incorporating generated tissue into a host where engineering-biology principles must be systematically applied via an interdisciplinary approach [10,11]. In addition, medical textile fiber and fabrics used in hygiene and healthcare (wound care, bandages, sutures, and meshes) were also actively researched [12]. Specifically, three-dimensional (3D) medical textiles can be employed in wound dressing, vascular grafting, and scaffolding for tissue engineering. It was included in the medical devices classification system established by the US Food and Drug Administration [13]. On the other hand, textile forming technologies such as two-dimensional (2D) weaving, 2D knitting, 2D braiding, three-dimensional (3D) weaving [14], and 3D braiding [15,16] were used to develop engineered 3D tissue scaffolds for various tissues and organs [17]. Further, textile fabric, substrate, or preform [18,19] in developing artificial organs has shown its unique advantages in mimicking human tissues’ hierarchical, anisotropic, and strain-stiffening properties [20]. Three-dimensional textile preforms architectures affect the fiber volume fraction [21], porosity [22], and mechanical properties of 3D composites [23,24,25]. Various textile patterns [20] and their mechanical properties need to be explored for tissue engineering to find textile-based scaffolds [26] that match the mechanical properties of their target tissues [27,28]. Future studies may be concentrated on scaffolds with different structures to induce cells and modeling the fiber-based scaffold to simulate mechano-chemical and mechano-biological properties considering natural organs [17,29]. 

Natural polymers employed for scaffold fabrication were considered protein-based (silk, fibrin, gelatin and collagen, and dextran) [30] and carbohydrate-based (agarose, alginate, hyaluronan and chitosan) in that they are generally hydrogels, which are three-dimensional cross-linked hydrophilic structures [31]. Contrarily, synthetic polymers (thermoplastic) in used 3D scaffolds for cartilage tissue engineering are the poly-α-hydroxy esters, especially polylactic acid (PLA), polyglycolic acid (–(C_2_H_2_O_2_)_n_–O–CO–CH_2_–, PGA) [32] and poly(lactide-co-glycolide) (PLGA) [33,34], which is formed as a continuous filamentary bundle structure (fiber or yarns) via melt spinning, wet spinning, and electrospinning. They have better mechanical properties and are naturally biodegradable fibers [35,36]. 

The effects of PLA/PGA composition on the physical adhesion and growth kinetics of chondrocytes seeded onto PLA/PGA nonwoven scaffold were studied. Altering the ratio of PLA to PGA caused insignificant differences in the scaffolds’ compressive moduli and degradation times and changes in adhesion, shape, and proliferation rates of cells seeded onto the hybrid scaffold [37]. Polylactic acid (PLA)-coated polyglycolic acid (PGA)/hydrogel scaffold was used for articular cartilage repair. It was found that scaffold stiffness, degradation time, cell seeding efficiency, and proliferation rates were the critical parameters, and they affected the engineered design of the extracellular matrix-based scaffold [38]. Polylactic acid (PLA) and polyglycolic acid (PGA) polymers were degraded by hydrolysis and enzymatic activity, which depended on critical parameters such as their molecular structure, crystallinity, and copolymer ratio [39]. They can be formed into porous scaffolds or carriers of cells, extracellular matrix components, and bioactive agents [39,40]. 

The in vitro degradation of PGA was followed by observing the mass, dimensions, crystallinity, tensile strength changes, and surface structure [41]. It was reported that PGA degradation was affected by polymer chain orientation, crystallinity, and immersion media, such as alkaline [41]. Additionally, tensile strength loss was due to chain scission in the amorphous regions, while mass loss appeared when polymer chains became small enough to be soluble [41]. Textured staple polyglycolic PGA fiber (diameter: 13 μm) was formed into a nonwoven fabric (thickness 5 mm) via the carding–needling process. It was obtained that cartilaginous tissue consisting of glycosaminoglycan and collagen was successfully regenerated in the shape of the original PGA scaffold [42]. It was revealed that poly(l-lactic acid) (PLLA) and poly(d,l-lactic-co-glycolic acid) (PLGA)-coated polyglycolic acid (PGA) fiber tubular structure has smooth muscle cells in vitro with appropriate cell distribution due to improved compression strength [43]. The major disadvantages of PLA such as its poor ductility, slow degradation rate, and poor hydrophilicity somewhat limit its applications [44]. Modifying of PLA bulk and surface properties has thus become crucial to increase its applicability [44]. 

On the other hand, chitosan is a polysaccharide-based natural biopolymer with valuable physico-chemical properties, particularly interactions with proteins, cells, and living organisms [45]. It can be used in 3D preform scaffolds [46]. Furthermore, nanoparticle (silica) or nanofiber-added hydrogels exhibited better mechanical strength properties in tissue engineering cartilage, enhancing cell growth and tissue regeneration due to the biomimetic properties of nanomaterials [35,47]. On the contrary, stem cells-based 3D biomimetic scaffold can work under a natural repair process [48] because it provides a simulated natural environment with chemical and contact guidance for stem cell differentiation [49]. Moreover, it was stated that topographic cues could effectively regulate various mechanisms including the adhesion [50], morphology, migration [51], differentiation [52], proliferation, apoptosis, and other behaviors of cells [53], a phenomenon known as contact guidance [54]. Under in vivo conditions, cells are guided by the complex topographic structures of the extracellular matrix (ECM) [48]. The recent advances in the field of regenerative medicine especially considering multifunctional biomaterials for building biomimetic scaffold in order to regenerate stem cells in vivo were provided [55]. 

A three-dimensional (3D) poly(lactic-co-glycolic acid)(PLGA)/poly (e-caprolactone) (PCL) scaffold based on wet-electrospinning [56] that consisted of a very loose, dispersive, and randomly oriented porous fiber structure for cell population and differentiation [57], and eventually for the generation of osteochondral (bone) tissue, was developed via the endochondral approach [58]. Layered natural endometrial stromal cells were cultured in a 3D PGA electrospun scaffold in which an electrospun nanoweb was overlaid by a monolayer of endometrial epithelial cells, and it was found that the overall arrangement was identical to the native endometrium [59]. 

Three-dimensional (3D) bioprinting is a method to create artificial multi-cellular tissues or organs in regenerative medicine and tissue engineering [60]. Three-dimensional bioprinting involves various techniques including droplet, thermal inkjet, acoustic inkjet, micro extrusion, laser-assisted, and stereolithography bioprinting [61,62]. An osteochondral scaffold for articular cartilage repair was created by using three-dimensional printing. The material composition, porosity, macro-architecture, and mechanical properties were varied throughout the scaffold structure [63]. 

Another study stated that flocked substrate with polyhydroxy butyrate (PHB), polylactide (PLA), chitosan, or collagen is an isotropic highly porous scaffold with high compressive strength and can be used to repair human organs such as cartilage and bone tissue as well as extra-corporeal organs because of adequate vascularization, mechanical strength, and the osseointegration of the scaffold [64].

Articular cartilage study, in particular osteoarthritis (degenerative joint disease), revealed that the parameters such as cell source, signaling molecules, scaffolds architecture [65,66], porosity [67,68,69], permeability and stiffness [70], extracellular matrix content [71], mechanical stimulation, and structure functionality are of paramount importance [72,73,74]. In this respect, biodegradable scaffolds in cartilage tissue engineering provide a comfortable environment for synthesizing cartilage matrix by host cells and temporarily replace the function of the native matrix until new cartilage has formed [75]. For these reasons, the scaffolds have biodegradable, non-toxic byproducts, a porosity that allows diffusion of nutrients and waste products [76], support cell viability, proliferation [77], differentiation [78], and extracellular matrix (ECM) production [78], integrated with the tissue at the defect site, and give mechanical support to the engineered tissue [72,73,79,80]. It was indicated that the scaffold architecture was pivotal in supporting the cellular entrapment within filamentary scaffold [81]. It was also found that scaffolds must have the adequate mechanical strength to provide structural integrity during new tissue regeneration in which the scaffold properties match the native tissues in vivo [82]. 

It was claimed that the entanglements between filaments and pores in scaffold architecture were crucial in supporting cellular bridging, promoting cellular migration, nutrient transportation, and tissue in-growth [83,84,85]. Furthermore, filament continuity provided the smooth shear stress flow along the inter- and intralayer in the scaffold during exerted biomechanical force [86]. The influence of void fraction and pore size of poly(-lactic acid) (L-PLA) scaffold on the attachment, growth, and extracellular matrix deposition by some cell types was studied. It was found that uniform seeding on scaffolds with a 90% void fraction for each pore size was achieved compared to the scaffold with a 75% void fraction. In addition, it was noted that structural consistencies and irregularities of the scaffold, including material clustering, pore size, surface area, wall thickness, and homogeneity are of critical importance [87]. A mechanoregulation theory based on scaffold shear strain and fluid shear stress was applied to determine scaffold architecture’s influence (pore volume and size) on the mechanical stimuli within initial conditions. Results indicated that shear stress distribution induced by fluid perfusion depends on pore distribution within the scaffold. Gyroid architectures provide better accessibility of the fluid than hexagonal structures [88]. These findings showed that the low permeability design with a spherical pore provided a better microenvironment for chondrogenesis (cartilage matrix) than the high permeability design with a cubical pore [89]. A 3D well-orthotropic woven scaffold/chondrocyte–hydrogel (agarose and fibrin) matrix cartilage tissue composite was developed. Its mechanical (tensile, compressive, and shear) properties were almost equal to natural articular cartilage [90]. The performance of 3D woven composites for a specific application including artificial organ development or organ repair, can be optimized by appropriately selecting the geometric parameters including fiber types, cross-section, and the preform architecture and directional fiber volume. The geometric modeling estimates the volume fraction of filamentary tows and areal density of preforms due to tow crimp in the preform architecture [91,92,93,94,95]. Many challenges remain in polymer-scaffold processing, including the fabrication of highly porous scaffolds of defined architecture to guide tissue growth, the manufacture of high-strength load-bearing scaffolds to replace hard tissues, and the incorporation and delivery of growth factors, and other tissue induction factors to stimulate or enhance organ regeneration [96]. 

In this study, biodegradable and biocompatible PGA fiber woven scaffolds were developed due to z-yarn reinforcement in the through-the-thickness of the structure. Compared to extrusion 3D printing and electrospinning, there is no true through-the-thickness fiber in the structures, which could affect the cell growth in the thickness during 3D organ development. The literature shows that biocompatible and biodegradable fiber, 3D fiber architecture, fiber volume fraction, pore geometry, and size are crucial for 3D scaffold development, especially for cartilage tissue material applications. Therefore, this research study aimed to develop 3D fiber architectures and experimentally determine their total and directional fiber volume fraction and porosity. Moreover, geometric modeling on fiber volume fraction and porosity was also provided, considering generated experimental data. 

## 2. Materials and Methods

### 2.1. Development of 3D Structures for Scaffold

Polyglycolic acid (PGA, %100) fiber (Meta Biomed Inc., Cheongju, Ch’ungch’ong-bukto, Republic of Korea, KR) was utilized for making three-dimensional woven preforms for possible scaffold applications. The PGA filament diameter was 13.7 micron. Yarn linear density and density were 6.22 tex and 1.56 g/cm^3^, respectively. Table 1 presents some of the critical specifications of polyglycolic acid fiber from open literatures [97,98]. PGA yarn was plied (50 yarns, 311 tex) to make sufficiently dense 3D woven preforms based on Kundak’s PhD study under supervision of Bilisik in Graduate School of Natural and Applied Sciences in Erciyes University [99]. Three types of 3D PGA fiber preform architectures based on a previous study of Bilisik et al. [19] were developed, called 3D plain woven preform (3DW-P-ZP), 3D semi-interlaced woven preform (3DW-P-ZO), and 3D orthogonal woven preforms (3DW-O-ZO). They are explained in the following subtitles. 

#### 2.1.1. 3D Plain Woven Preform

**Figure 1:**
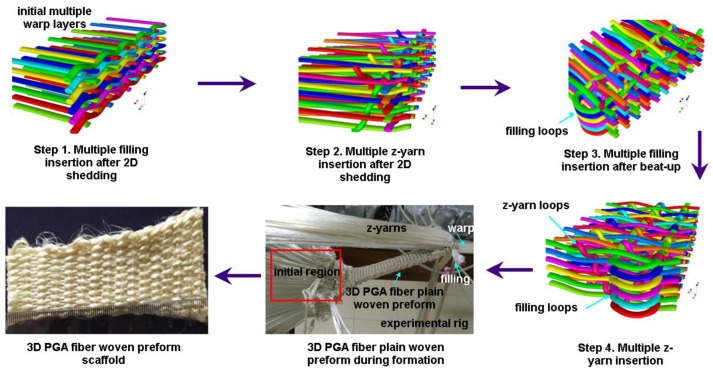
Three-dimensional plain (3DW-P-ZP) preform scaffold and textile bonding steps incorporated with schematic unit cell (TexGen) during formation via an experimental rig (digital image).

An experimental rig was refined to make a 3D completely interlaced plain woven preform (warp/filling/z-fiber, plain), semi-interlaced woven preform (warp/filling plain, z-fiber orthogonal), and 3D orthogonal woven preforms (warp/filling/z-yarn, orthogonal) in Nano/Micro Fiber Preform Design and Composite Laboratory in Erciyes University (TR). This method generally involves six distinctive steps to create six-layer preforms, including warp let-off, two-dimensional (2D) shedding or one-dimensional (1D) shedding, multiple filling insertions, z-yarn insertion, beat-up, and take-up [19]. Figure 1 shows the 3D plain weave steps incorporated with schematic unit cell (TexGen) and actual preform. The 3D plain woven preforms can be defined as warp–filling and warp–z-yarns interlaced based on plain (1/1/1) weave pattern to generate filamentary structure [19]. Woven interlacement can be called textile bonding, an analogy from chemical bonding. It is a unique bonding mechanism interconnecting filamentary bundle (TOW) to create architectural structures for specific requirements. After the individual warps of 3D PGA fiber plain woven preform (3DW-P-ZP) were aligned in a matrix of rows and columns as an initial step, an even number of warp layers in the column direction were sequentially moved by using the 2D shedding. Then, fillings between each warp layer in the row direction were inserted for putting the fiber transversely across the in-plane direction before z-yarns were inserted between each warp layer in the column direction for putting the fiber through-the-thickness of the preform. After inserted fibers were beaten up towards the preform formation line, an even number of warp layers in the row direction were sequentially moved by using the 2D shedding. Again, fillings between each warp layer in the row direction were inserted before z-yarn insertion was completed. The last step was take-up to remove the preform from the formation zone. These steps were repeated depending on the required preform length (Figure 1).

#### 2.1.2. 3D Semi-Interlaced Woven Preforms

**Figure 2:**
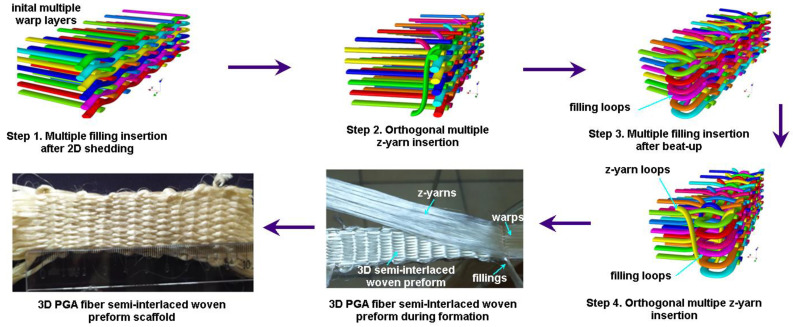
Three-dimensional semi-interlaced (3DW-P-ZO) preform scaffold and textile bonding steps incorporated with schematic unit cell (TexGen) during formation via an experimental rig (digital image).

The 3D semi-interlaced woven preform was achieved by warp and weft, interlaced based on a plain pattern before warp, and z-yarns were interlaced depending upon an orthogonal pattern (warp/filling plain, z-yarn orthogonal) [19]. Figure 2 shows the 3D semi-interlaced weave steps incorporated with schematic unit cell (TexGen) and preform. After the individual warps of 3D PGA fiber semi-interlace woven preform (3DW-P-ZO) were aligned in a matrix of rows and columns as an initial step, an even number of warp layers in the column direction were sequentially moved by using the 1D shedding. Then, fillings between each warp layer in the row direction were inserted for putting the fiber transversely in the in-plane direction before z-yarns were orthogonally inserted between each warp layer in the column direction for putting the fiber through-the-thickness of the preform. After inserted fibers were beaten up towards the preform formation line, an even number of warp layers in the column direction were sequentially and reversely moved by using 1D shedding. Again, fillings between each warp layer in the row direction were inserted before z-yarns insertion was orthogonally completed. The last step was take-up to remove the preform from the formation zone [19]. These steps were repeated depending on the required preform length (Figure 2).

#### 2.1.3. 3D Orthogonal Woven Preforms

**Figure 3:**
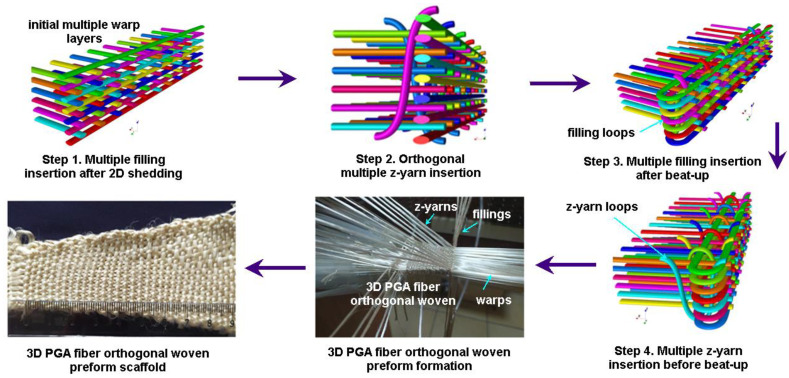
Three-dimensional orthogonal (3DW-O-ZO) preform scaffold and textile bonding steps incorporated with schematic unit cell (TexGen) during formation via an experimental rig (digital image).

The 3D orthogonal woven preform can be made using warp, filling, and z-yarn, which were orthogonally formed in that interlacement that occurred at the surfaces of the preform (warp/filling/z-yarn orthogonal) [19]. Figure 3 shows the 3D orthogonal weave steps incorporated with schematic unit cell (TexGen) and preform. The warp was arranged in a matrix of rows and columns to make 3D PGA fiber orthogonal woven preform (3DW-O-ZO). The first step was filling insertion between each warp layer in the row direction. The second and third steps were z-yarn insertion between each warp layer in the column direction without any interlacement and beat-up, respectively. The fourth step was take-up [19]. After the above steps were repeated, the 3D PGA fiber orthogonal preform structure was made (Figure 3). 

### 2.2. Dimensions, Density, and Angle Measurements

Measurements of all 3D woven preforms (3DW-P-ZP, 3DW-P-ZO, 3DW-O-ZO) were accomplished in normal conditions (out-of-loom). The measurements of the preforms were executed in force-free environments [99]. Structure length (*S_l_*), width (*S_w_*), and thickness (S_t_) measurements of the 3D PGA fiber woven preform structures were fulfilled. The precision of dimensional measurements was 0.01 mm. On the other hand, length, width, and thickness measurements were performed on the different parts of the 3D woven preform structures and were repeated three times. Yarn-to-yarn distance on warp/warp (w-w), filling/filling (f-f), and z-yarn/z-yarn (z-z) in the top, side, and cross-sections of all 3D woven structures were determined. Measured yarn lengths in all woven preform structures included uncrimped warp length (*l_w_*), uncrimped filling length (*l_f_*), uncrimped total z-yarn length (*l_zt_*), z-yarn arc length in the structure surface (*l_za_*), and z-yarn length in the structure thickness (*l_z_*). The precision of yarn length measurements was 0.2 mm. The densities of the warp, filling, and z-yarn were identified on the top, side, and cross-section of the 3D PGA fiber woven preform. The precision of density measurements was 0.2 ends/10 mm. Measured angles on all 3D PGA fiber woven preform structures were defined as follows: θ_wc_ was the warp angle in the fabric thickness; θ_wm_ was the warp angle in the fabric width; θ_ws_ was the warp angle in the fabric length; θ_fc_ was the filling angle in the fabric thickness; θ_fm_ was the filling angle in the fabric width; θ_fs_ was the filling angle in the fabric length; θ_zc_ was the z-yarn angle in the fabric thickness; θ_zm_ was the z-yarn angle in the fabric width; θ_zs_ was the z-yarn angle in the fabric length. The precision of angle measurements was 0.2 degrees. The dimensional measurements of all 3D woven preforms were performed using the Newman digital caliper (measurement length: 0–150 mm and precision: 0.01 mm, China). The uncrimped yarn length and arc-length measurements were performed using a flexible ruler (0–300 mm measurement length and precision 0.2 mm). The angle measurements were performed using the manual angle instrument (measurement angle: 0–180° and precision: 0.2 degrees). 

#### 2.2.1. Crimp Measurements

Crimps in the 3D plain and semi-interlaced woven preforms were calculated based on the measured structure dimensions and the uncrimped yarn lengths considering a Tautex digital instrument (James H Heal Co., Halifax, UK) principle. For this reason, the following Equations (1)–(3) were employed [19].

(1) $$C_{w}(\%)=\frac{\left(l_{w}-S_{l}\right)}{S_{l}}\times 100$$

(2) $$C_{f}(\%)=\frac{\left(l_{f}-S_{w}\right)}{S_{w}}\times 100$$

(3) $$C_{z}(\%)=\frac{\left(l_{zt}-S_{t}\right)}{S_{t}}\times 100$$

where *C_w_* is the warp crimp (%), *l_w_* is the uncrimped warp length (mm), *S_l_* is the structure length (mm), *C_f_* is the filling crimp (%), *l_f_* is the uncrimped filling length (mm), *S_w_* is the structure width (mm), *C_z_* is the z-yarn crimp (%), *l_z_*_t_ is the uncrimped total z-yarn length (mm), and *S_t_* is the structure thickness (mm). 

#### 2.2.2. Fiber Volume Fraction Measurements

Fiber volume fraction and void fraction (porosity) were measured considering ASTM D3171-99 and ASTM D2734-91 standards. The volume-based fiber volume fractions of all 3D PGA fiber woven preforms were then calculated by using Equations (4)–(7) [100,101].

(4) $$V_{fp}=\frac{\rho_{p}}{\rho_{f}}100$$

(5) $$V_{fw}(\%)=\left(\frac{M_{w}}{M_{p}}100\right)\frac{\rho_{p}}{\rho_{f}}$$

(6) $$V_{ff}(\%)=\left(\frac{M_{f}}{M_{p}}100\right)\frac{\rho_{p}}{\rho_{f}}$$

(7) $$V_{fz}(\%)=\left(\frac{M_{z}}{M_{p}}100\right)\frac{\rho_{p}}{\rho_{f}}$$

where *V_fp_* is the total fiber volume fraction of 3D woven preforms (%), *ρ_p_* is the fiber density of 3D woven preforms (g/cm^3^), *ρ_p_* is the PGA fiber density (g/cm^3^), *V_fw_* is the fiber volume fractions of warp yarns (%), *V_ff_* is the fiber volume fractions of filling yarns (%), and *V_fz_* is the fiber volume fractions of z-yarns (%), *M_w_* is the total mass of warp yarns (gram, g), *M_p_* is the total mass of preform (g), *M_f_* is the total mass of weft yarns (g), and M_z_ is the total mass of z-yarns (g). 

In addition, all 3D PGA fiber preform structures were examined by an optical microscope (Olympus SZ61-TR equipped with Bs200DOC digital image analysis software). A high-resolution digital camera (CANON PowerShot SX30 IS, Tokyo, Japan) was used to image the surface of the PGA fiber structures. Weight measurements were performed on all 3D preform structures using an Ohaus Adventurer^TM^ Pro AV812 (Ohaus Corp., Parsippany, NJ, USA) digital balance. The error in the measurement of weight was ±0.1 mg. All tests were conducted in the standard laboratory atmosphere having a temperature of 23 °C ± 2 °C and relative humidity of 50% ± 10% [102]. 

### 2.3. Geometric Modeling

#### 2.3.1. Analytical Model on Volume Fraction and Porosity

Analytical relations on fiber volume fraction and porosity (void content) were proposed for 3D PGA fiber plain woven (3DW-P-ZP), 3D semi-interlaced woven (3DW-P-ZO), and 3D orthogonal woven structures (3DW-O-ZO). Table 2 presents fiber volume fraction equations for 3D plain woven preform. Table 3 presents fiber volume fraction equations for 3D semi-interlaced woven preform and Table 4 shows fiber volume fraction equations for 3D orthogonal woven preform. The analytical models are generally developed based on meso-scale ideal representative volume element from produced actual unit cell of each 3D PGA fiber woven preform structure. Thus, it was assumed that warp, filling, and z-yarn diameters in the 3D preform structure remained circular. Inter-yarn compression and frictional inter-filamentary migrations were neglected. The interlaced and noninterlaced regions in the 3D structures were homogeneously created during the formation process [103]. The representative unit cell geometry (representative volume element) was uniformly displaced and consistently formed throughout the 3D woven structure. Equations (8)–(12) displace fiber volume fraction equations for 3D plain woven preform. Further, Equations (13)–(15) displace fiber volume fraction equations for 3D semi-interlaced woven preform, and Equations (16)–(18) displace fiber volume fraction equations for 3D orthogonal woven preform. 

#### 2.3.2. Finite Element Modeling (FEM) on Volume Fraction and Porosity (TexGen)

We used TexGen version 3.12.1 software to model the fiber volume fraction and porosity. The University of Nottingham developed TexGen software for modeling the geometry of textile structures where yarn can be considered various geometric shapes such as circular, ellipse, lenticular, or interpolated cross-sections. Only the smallest repeatable section of yarns was described. However, it can be combined with the repeat vectors depending on the almost infinite meso-scale fabric boundary. Therefore, it is necessary to restrict the model to a finite region of space called the domain. In most cases, the domain corresponds to the unit cell of the textile structure. For instance, after the fabric unit cell was defined considering meso-level design, fiber volume fraction and porosity were computed (*V_f_* = *A_f_*/domain, defined unit cell area). TexGen models may be used as the basis of simulations to predict various properties, including textile geometry and mechanics, permeability, and composite mechanical behaviors. Release TexGen software included an update to octree voxel mesh refinement such that an initial number of voxels can be specified, the addition of octree refinement option to export ABAQUS voxel files in the graphical user interface (GUI), correct small intersections tool and various minor changes and bug fixes [104,105,106,107,108].

### 2.4. Stiffness Calculations on 3D Preforms

Preliminary stiffness and partly strength (tensile and shear) properties of all developed 3D PGA fiber preform structures for scaffolding were theoretically identified considering micromechanics relations as shown in Equations (19)–(21) [109]. Therefore, idealized PGA fiber preform/matrix scaffolds were macroscopically defined and compared to the cartilage. It was assumed that the fiber in preform for the scaffold was homogeneous, linearly elastic, isotropic, regularly spaced, perfectly aligned, and bonded with matrix (hydrogel). In addition, the matrix was homogeneous, isotropic, and linearly elastic. No voids can exist between fiber and matrix. On the other hand, all 3D preforms were initially stress-free, linearly elastic, macroscopically homogeneous, and well-orthotropic [109]. Thus, micromechanical relations (19)–(21) can be applicable on 3D preform scaffolds. All stiffnesses and strength properties of all 3D woven preforms were computed via measured fiber volume fractions, fiber volume fraction derived from analytical relations, and numerical model (TexGen). Micromechanical relations for 3D preform scaffold were provided in Equations (19)–(21) [109]. Using the micromechanical Equations (19)–(21), we can predict the scaffold directional tensile modulus (*E*), shear modulus (*G*), and Poisson ratios (*ν*) properties.

(19) $$E_{p}=E_{f}\times V_{fp}+E_{m}\times V_{mp}$$

(20) $$G_{p}=G_{f}\times V_{fp}+G_{m}\times V_{mp}$$

(21) $$\nu_{p}=\nu_{f}\times V_{fp}+\nu_{m}\times V_{mp}$$

where *E_f_* is the fiber modulus; *E_m_* is the matrix modulus; *V_fp_* is the preform fiber volume fraction; *V_mp_* is the preform matrix volume fraction; *E_p_* is the preform modulus; *G_f_* is the fiber shear modulus; *G_m_* is the matrix shear modulus; *G_p_* is the preform shear modulus; *ν_f_* is the fiber Poisson ratio; *ν_m_* is the matrix Poisson ratio; *ν_p_* is the preform Poisson’s ratio.

In addition, the fiber strain was presumed equal to the matrix strain in warp, filling, and z-yarn direction. Therefore, strength Equation (22) can be accepted for defining the directional and total tensile strength of 3D PGA fiber preform structure for scaffold critical volume fractions were achieved via proper preform architecture designs as explained in preform fabrications (Figure 1, Figure 2 and Figure 3). Residual effects, including strength (tensile, shear) and Poisson (directional deformation) parameters between warp/filling and warp/z-yarn were not considered during tensile strength calculations. Moreover, fiber-to-fiber interlacement (textile bonding or filamentary entanglement) in all 3D preforms, which caused stress concentration (straining), were neglected. So, the strength properties of all 3D preform scaffolds can be identified using Equation (22) [109].

(22) $$\sigma_{ps}=\sigma_{f}\times V_{fp}+\sigma_{m}\times V_{mp}$$

where *σ_f_* is the fiber tensile strength; *σ_m_* is the matrix tensile strength; *σ*_ps_ is the preform tensile strength.

## 3. Results and Discussion

### 3.1. Preform Structure for Scaffold Results

**Figure 4:**
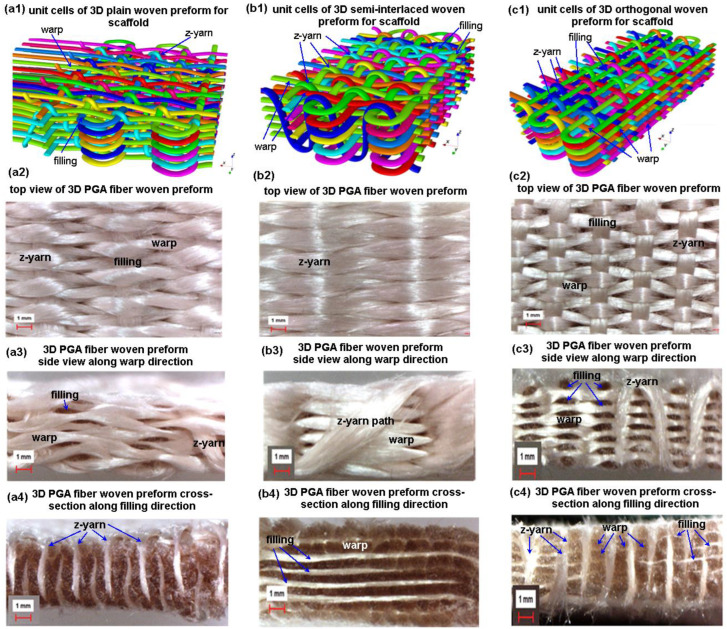
Three-dimensional woven preform unit cells. (**a1**–**a4**) 3D plain (3DW-P-ZP) with schematic unit cell (TexGen), top, side, and cross-section of microstructure images (optical microscope, ×6.7), respectively; (**b1**–**b4**) 3D semi-interlaced (3DW-P-ZO) with schematic unit cell (TexGen), top, side, and cross-section of microstructure images (optical microscope, ×6.7), respectively; (**c1**–**c4**) 3D orthogonal (3DW-O-ZO) with schematic unit cell (TexGen), top, side, and cross-section of microstructure images (optical microscope, ×6.7).

Three-dimensional PGA fiber plain (1/1) woven preform for scaffold has interlacements in-plane and out-of-plane of the preform. This interlacement was created between warp-filling in the plane and warp-z-fiber in the out-of-plane during formation as shown in Figure 4(a1–a4). This caused coarse structure, resulting in sizeable local porosity around the fiber interlacement boundary (Figure 4(a2–a4)). Probably, this could be useful for cell adhesion and migration of cells through the predetermined filamentary TOW path to provide mechanical stimulation during cell growth and cell proliferation, especially 3D organ development. On the other hand, polyglycolic acid fiber is biocompatible, biodegradable, and well suited for 3D preform scaffold applications. Thus, it is probably formed three-dimensionally, especially the insertion of the z-yarn in the through-the-thickness of the scaffold to improve cell adhesion and growth. PGA fiber is a continuous form on the surface and inside of the preform (meso-scale) (Figure 4(a3,a4)). In addition, 3D preform surface topography can be tailored depending on cell distribution in the directional fiber volume fraction and fiber directions [9]. This proof-of-concept study was successfully demonstrated for its feasibility. Nonetheless, in vitro and in vivo studies should be carried out on the 3D developed PGA scaffold for future research endeavor. To manufacture higher-order interlaced 3D PGA fiber preform from the loom, two-dimensional shedding must be used and the loom should be automated for consistent preform fabrication for scaffolding for tissue engineering. 

Three-dimensional PGA fiber semi-interlaced woven preform for scaffold has interlacements in-plane (plain 1/1), and no interlacements (orthogonal) were out-of-plane of the preform. This 3D structure was made between warp-filling in the plain (1/1) pattern in the plane, and warp-z-fiber in the out-of-plane was orthogonally formed, as shown in Figure 4(b1–b4). Therefore, directional porosity, fiber volume fraction and predetermined PGA fiber path were crated differently (Figure 4(b2–b4). Perhaps this could influence cell distribution and cell differentiation, including cell contact guidance, which means, regulating the adhesion of cells, cell morphology, migration and cell proliferation [50,51]. On the other hand, 3D PGA fiber orthogonal woven preform for scaffold has no interlacements in-plane and out-of-plane of the preform except preform surface where fibers were high order curvature arc form as exhibited in Figure 4(c1–c4). Therefore, uniform fiber volume fraction and porosity can comparatively achieve (Figure 4(c2–c4)). This perhaps affects cell differentiation mainly, including cell contact guidance phenomena. However, in vitro and in vivo studies on all developed 3D PGA fiber preforms for scaffolds are required.

#### 3.1.1. Preform Fiber-to-Fiber Space, Density, and Areal Density Results

**Figure 5:**
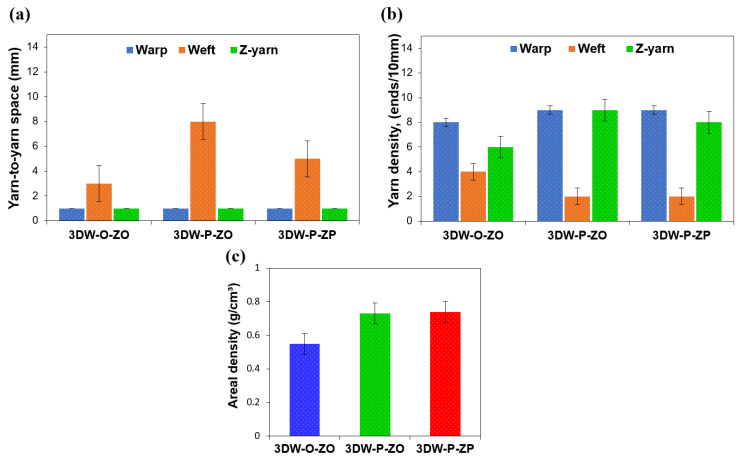
(**a**) Yarn-to-yarn space in the top of the 3D PGA fiber woven preform structures; (**b**) density in the top of all 3D woven preform structures, and (**c**) areal density of all 3D woven preform structures.

Yarn-to-yarn space, density, and angles of all 3D PGA fiber preform structures are presented in Table 5. Figure 5a–c illustrates yarn-to-yarn space, density and areal density of the 3D PGA fiber woven preform structures. Table 5 and Figure 5a show that warp-warp spaces in all 3D structures were almost uniform. However, filling-filling spaces in 3DW-P-ZO were 60% and 14.29% larger at the top of 3DW-P-ZP and 3DW-O-ZO, respectively. On the other hand, the filling-filling space on the side of all 3D structures was slightly distorted during filling insertion, probably due to the low bending rigidity of PGA fiber and process parameters, including light beat-up and interlacement-based preform architecture. The filling-filling distance of 3DW-P-ZO was over twice as large as the 3DW-P-ZP and 3DW-O-ZO due to in-plane and out-of-plane fiber interlacement differences. Z-yarn–z-yarn distances of 3DW-P-ZO and 3DW-P-ZP were 66.67% and 83.83% larger than the 3DW-O-ZO structure. The reason for the z-yarn–z-yarn distance differences between structures on the side were mainly through-the-thickness interlacement patterns (textile bonding) in that it resisted z-yarn during formation after z-yarn insertion. As seen in Table 5 and Figure 5b, preform density (ends/10mm) of warp in all 3D structures was approximately homogenous. However, the filling density of 3DW-O-ZO was twice as compared to the top of 3DW-P-ZP and 3DW-P-ZO. This was because of orthogonal fiber placement in the preform structure. In addition, z-yarn densities on the side of 3DW-P-ZP and 3DW-P-ZO were twice less dense than 3DW-O-ZO due to especially in-plane interlacements, which created frictional resistance during z-yarn packing in the formation zone. Additionally, the preform areal densities of the 3DW-P-ZP and 3DW-P-ZO structures were about 34.55% higher than 3DW-O-ZO, as exhibited in Figure 5c.

#### 3.1.2. Preform Structure Angle Results

**Figure 6:**
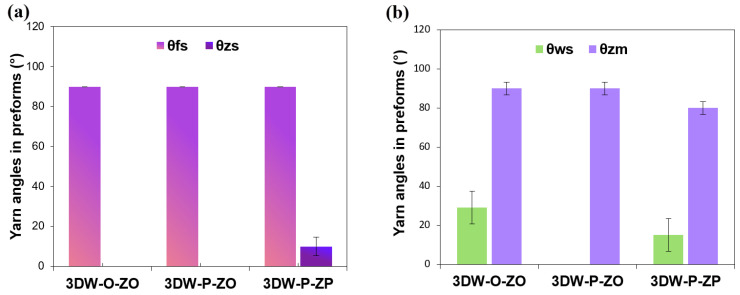
(**a**) Angle of the warp along the length and angle of the z-yarns along the width of the 3D preform structures; (**b**) angle of weft and z-yarns along the length of the 3D PGA fiber preform structure.

Figure 6a,b exhibits angles of warp, weft, and z-yarn along the length of the 3D PGA fiber preform structures. As seen in Table 5 and Figure 6a, the warp angle in the preform length (θ_ws_) of 3DW-O-ZO was almost twice higher than 3DW-P-ZP, whereas the z-yarn made an angle (θ_zm_) in the fabric width of 3DW-P-ZP due to in-plane interlacement. On the other hand, the filling angle (θ_fs_) in the preform length was almost the same for all 3D PGA fiber preform structures and the z-yarn angle in the fabric length (θ_zs_) of 3DW-P-ZP was 10° as shown in Figure 6b. Probably, directional fiber angles in the structure potentially affect the cell density and 3D cell growth morphology. 

#### 3.1.3. Preform Structure Crimp and Fiber Length Results

**Figure 7:**
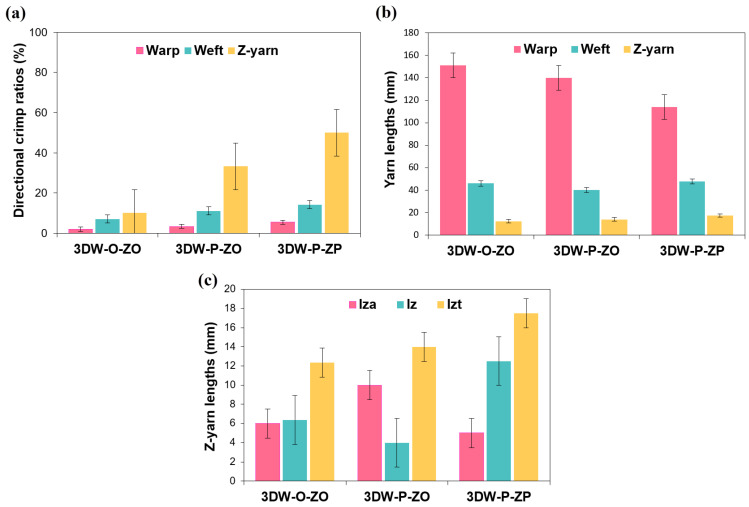
(**a**) Directional crimp of all 3D PGA fiber woven preform structure; (**b**) yarn lengths in the 3D PGA fiber woven preforms; (**c**) z-yarn length in the 3D PGA fiber woven preforms.

Crimp and fiber length measurement results of all 3D PGA fiber-woven preforms are presented in Table 6. Figure 7a–c shows directional crimp ratios, yarn length, and z-yarn length in all 3D PGA fiber woven preform structures. As seen in Table 6 and Figure 7a, directional crimp ratios were proportional, and the z-yarn crimp ratio (%) was the highest and followed by weft and warp crimp ratios (%). Z-yarn crimp (C_z_) in 3DW-P-ZP was 50% and five times greater than 3DW-P-ZO and 3DW-O-ZO, respectively. However, weft crimp (C_f_) in 3DW-P-ZP was 28.53% and 100% higher than 3DW-P-ZO and 3DW-O-ZO. In addition, warp crimp (C_w_) in 3DW-P-ZP was 61.34% and 177.5% larger than 3DW-P-ZO and 3DW-O-ZO. The weft yarn set obtained the lowest crimp ratios of all 3D structures. Z-yarn in all scaffold structures exhibited large crimp deviations due to out-of-plane and surface interlacement, which led to resistance during fiber compression. Furthermore, the large amounts of directional crimps in 3DW-P-ZP were received due to in-plane and through-the-thickness interlacement making the yarn sets more curvature. This could be beneficial during cell contact guidance, including cell growth rate and proliferation. 

As seen in Table 6 and Figure 7b, it was identified that yarn set lengths were proportional, and warp length was the longest, followed by filling and z-yarn lengths. The reason was that the warp in the surface of 3DW-O-ZO made a large arc compared to 3DW-P-ZP and 3DW-P-ZO. Furthermore, the total z-yarn length (l_zt_) in 3D-P-ZP was 25.10% and 41.59% lengthy compared to 3DW-P-ZO and 3DW-O-ZO, respectively (Figure 7c). This was because of the through-the-thickness yarn length (l_z_) in the 3D PGA fiber plain preform structure. This could be considered especially meaningful for making the 3D tissue formation because cells in the thickness of the scaffold can be growing via through-the-thickness fiber (z-yarn), including various possible mechanisms such as mechanobio stimulus effects, cellular migration, and cellular bridging during in-growth of tissue towards the thickness of scaffold. 

### 3.2. Volume Fraction and Porosity Results

**Figure 8:**
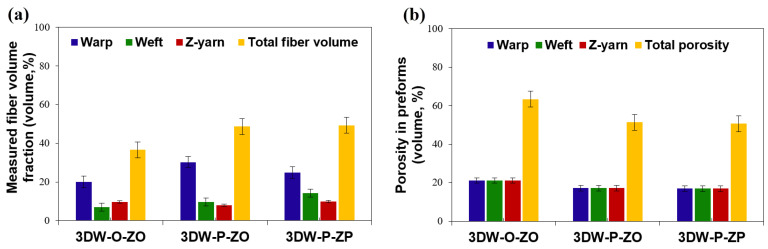
(**a**) Measured fiber volume fractions (%) and (**b**) fiber porosity fractions (%) of all 3D PGA fiber woven preform structures.

**Figure 9:**
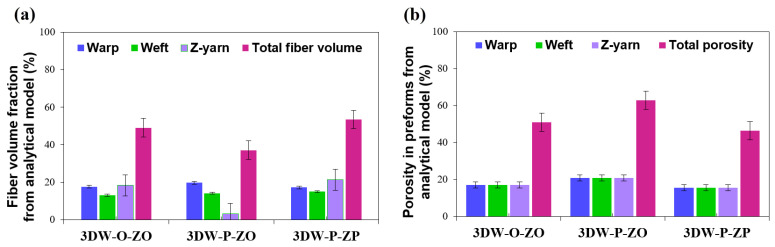
(**a**) Directional and total fiber volume fraction (%) and (**b**) directional and total fiber porosity (%) fractions of all 3D woven preform received from analytic relations.

**Figure 10:**
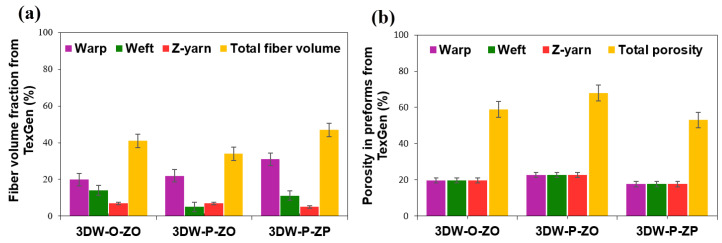
(**a**) Directional and total fiber volume fraction (%) and (**b**) directional and total fiber porosity (%) fractions of all 3D woven preform received from the numerical model (TexGen).

Fiber volume fractions based on measured values, analytic modeling, and numerical model (TexGen) of all 3D PGA fiber woven preforms are given in Table 7. Figure 8a,b shows fiber volume fractions (%) and fiber porosity fractions (%) of all 3D PGA fiber woven preform structures. Figure 9a,b illustrates the directional and total fiber volume fraction, and porosity fraction (%) of all 3D woven preform received from analytic relations. Figure 10a,b exhibits directional and total fiber volume fraction (%) and porosity fractions (%) of all 3D woven preform received from the numerical model (TexGen).

As seen in Table 7 and Figure 8a, measured directional and total volume fractions were proportional and total volume fraction of 3DW-P-ZP was slightly higher than 3DW-P-ZO and 3DW-O-ZO preform structures. They (*V_fp_*) were varied between 36.66% and 49.33%. In addition, warp fiber volume fraction was the highest, followed by filling and z-yarn. The *V_fw_* of 3DW-P-ZP was 17.76% lower than 3DW-P-ZO but 23.98% higher than 3DW-O-ZO. On the other hand, the *V_ff_* of 3DW-P-ZP was 45.73% and 101.28% greater than those of 3DW-P-ZO and 3DW-O-ZO. *V_fz_* of 3DW-P-ZP was 24.03% and 4.29% greater than those of 3DW-P-ZO and 3DW-O-ZO. As seen in Table 7 and Figure 8b, measured directional and the total porosity fractions (*V_tpr_*) were proportional and varied between 50.67% and 63.34%. In addition, total porosity fraction of 3DW-P-ZP was 1.31% and 20% lower than 3DW-P-ZO and 3DW-O-ZO preform structures, respectively.

Table 7 and Figure 9a show that the analytic model’s directional and total volume fractions were slightly proportional. The total volume fraction of 3DW-P-ZP was hardly higher than 3DW-P-ZO and 3DW-O-ZO preform scaffolds. They (*V_fp_*) varied between 37.08% and 53.52%. In addition, warp fiber volume fraction was mainly the highest and almost followed by filling and z-yarn, which was somewhat fluctuated probably due to thickness variations considering surface topography. *V_fw_* of 3DW-P-ZP was 12.63% and 2.49% low compared to 3DW-P-ZO and 3DW-O-ZO. On the other hand, the *V_ff_* of 3DW-P-ZP was 5.78% and 14.29% greater than those of 3DW-P-ZO and 3DW-O-ZO, respectively. *V_fz_* of 3DW-P-ZP was over five times and 14.29% greater than those of 3DW-P-ZO and 3DW-O-ZO, respectively. As seen in Table 7 and Figure 9b, directional and total porosity fractions (*V_tpr_*) were proportional and varied between 46.48% and 62.92%. In addition, the total porosity fraction of 3DW-P-ZP was 26.13% and 8.72% lower than 3DW-P-ZO and 3DW-O-ZO preform structures, respectively.

As seen in Table 7 and Figure 10a, directional and total volume fractions from the numerical model (TexGen) were about proportional. The total volume fraction of 3DW-P-ZP was slightly higher than that of 3DW-P-ZO and 3DW-O-ZO preform scaffolds. They (*V_fp_*) varied between 34% and 47%. In addition, the warp fiber volume fraction was the highest followed by filling and z-yarn. *V_fw_* of 3DW-P-ZP was 40.80% and 55% greater than those of 3DW-P-ZO and 3DW-O-ZO, respectively. On the other hand, the *V_ff_* of 3DW-P-ZP was 124% greater than 3DW-P-ZO and 20% lower than 3DW-O-ZO. *V_fz_* of 3DW-P-ZP was 28.57% lower than those of 3DW-P-ZO and 3DW-O-ZO. As seen in Table 7 and Figure 10b, directional and total porosity fractions (*V_tpr_*) were proportional and varied between 53% and 68%. In addition, the total porosity fraction of 3DW-P-ZP was 22.06% and 10.17% lower than 3DW-P-ZO and 3DW-O-ZO scaffold structures, respectively. This result may be considered that cell growth and cell density are perhaps proportional with directional fiber volume fractions for each preform scaffolds. 

#### Comparison of Measured Data, Analytic Model, and FE Model (TexGen)

**Figure 11:**
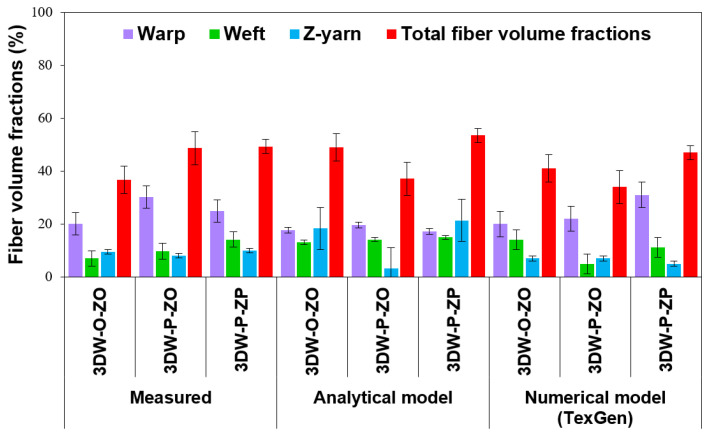
Directional and total fiber volume fractions of all 3D woven preform structures from measured data, analytic relations, and numerical model (TexGen).

Figure 11 shows directional and total fiber volume fractions of all 3D woven preform structures from measured data, analytic relations, and numerical model (TexGen). As seen in Table 7 and Figure 11, total volume fractions (*V_fp_*) of 3DW-P-ZP predicted by the analytic model were slightly differed (8.49% higher) compared to the measured value, whereas *V_fp_* predicted by the numerical model was 4.72% low compared to the measured fiber volume fraction. On the other hand, total volume fractions (*V_fp_*) of 3DW-P-ZO from the analytic model substantially differed (23.80% lower) compared to the measured value. In contrast, *V_fp_* from the numerical model was 30.13% lower than the measured fiber volume fraction. Furthermore, total volume fractions (*V_fp_*) of 3DW-O-ZO predicted by the analytic model were considerably differed (33.88% higher) compared to the measured value, whereas *V_fp_* predicted by numerical model was slightly 11.84% high compared to the measured fiber volume fraction. The differences in preform fiber volume fraction between the analytical, FE model, and measured valued attributed to the warp, filling, z-yarn (fiber TOW) cross-section deformation under inter-yarn pressure created during formation under constant tension where yarn (fiber TOW) cross-section was distorted from circular to high order degree ellipse or lenticular shape. This influenced fiber TOW porosity (or void) and created additional porosity around the interlacement section of the yarn sets. Therefore, preform scaffold dimensions were changed, especially thickness and width. Moreover, dimensional changes of in preforms can also affect their failure mechanism during the degradation period of PGA fiber in the scaffold architecture. 

Total porosity fractions (*V_prt_*) of 3DW-P-ZP predicted by analytic and numerical models were 7.87% and 4.60% higher than compared to the measured value, respectively. Additionally, total porosity fractions (*V_prt_*) of 3DW-P-ZO from analytic and FE models (TexGen) were substantially lower at 22.56% and 32.45% compared to the measured value. Further, the total porosity fractions (*V_prt_*) of 3DW-O-ZO predicted by analytic and TexGen models were 19.61% and 6.85% low compared to the measured value. The results demonstrated that volume fraction and porosity fractions of all 3D woven scaffolds could be accurately predicted depending upon biodegradable tissue regeneration requirements.

### 3.3. Stiffness and Strength on 3D Preforms Results

**Figure 12:**
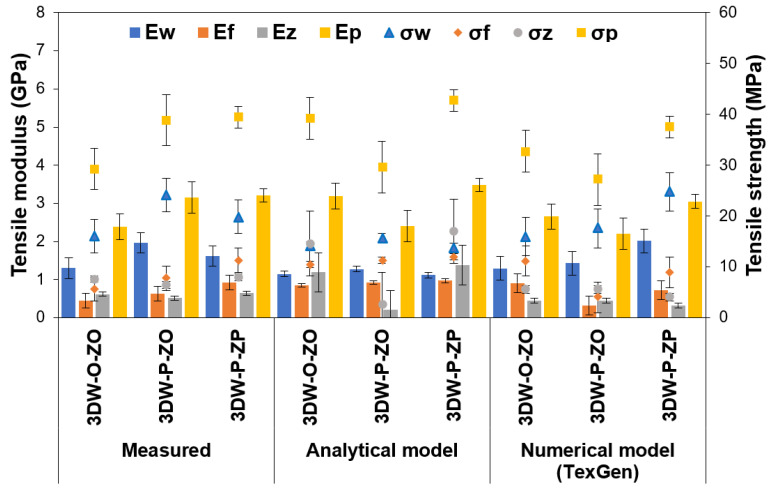
Directional and total preform stiffness and tensile strengths of all 3D PGA fiber preform scaffold from measured values, analytical model, and FE model (TexGen).

The stiffness and strengths of 3D PGA fiber preform scaffolds based on measured values, analytic model, and numerical model (TexGen) are presented in Table 8, where E_warp_ (E_11_), E_filling_ (E_22_), and E_z-yarn_ (E_33_) are the warp, filling, and z-yarn direction tensile modulus, respectively. Further, G_warp-filling_ (G_12_), G_filling-z-yarn_ (G_23_), and G_z-yarn-warp_ (G_13_) are the warp, filling, and z-yarn direction shear modulus, respectively. ν_warp-filling_ (ν_12_), ν_filling-z-yarn_ (ν_23_) and ν_z-yarn-warp_ (ν_31_) are the warp, filling, and z-yarn direction Poisson ratios, respectively. In addition, σ_warp_ (σ_11_), σ_filling_ (σ_22_), and σ_z-yarn_ (σ_33_) are the warp, filling, and z-yarn direction tensile strengths. These directional stiffness and strength calculations on 3D preforms were carried out considering micromechanical relations as explained in Section 2.4. Figure 12 illustrates directional and preform stiffnesses and tensile strengths of all 3D PGA fiber preform scaffolds from measured values, analytic model, and FE model (TexGen), and Figure 12 also exhibits tensile and shear modulus of all 3D PGA fiber preform scaffolds from measured values, analytic model, and FE model (TexGen). 

During stiffness and strength calculations, polyglycolic acid fiber tensile modulus and strength (average 6.5 GPa for modulus, 79.85 MPa for tensile strength) [98] and hydrogel tensile modulus and strength (average 75 × 10^−6^ GPa for modulus, 25 × 10^−3^ for tensile strength) [110,111] were considered. In addition, polyglycolic acid fiber Poisson ratios (average 0.35) [112,113] and hydrogel Poisson ratios (average 0.457) [113,114] were conceded. Moreover, directional and total fiber volume fractions of the 3DW-P-ZP, 3DW-P-ZO, and 3DW-O-ZO preform scaffold defined by measured values, an analytical model, and a numerical model (TexGen) were utilized, and a micromechanics principle was applied via tensile modulus Equation (19), shear modulus Equation (20), Poisson ratio Equation (21), and tensile strength Equation (22). 

As seen in Table 8 and Figure 12, the tensile modulus (E_p_) of 3DW-P-ZP was the highest value, followed by 3DW-P-ZO and 3DW-O-ZO structures. In addition, the warp modulus (E_warp_) was the highest compared to the filling (E_filling_) and z-yarn (E_z-yarn_) modulus. The tensile moduli obtained from measured, analytic, and numerical models were close to each other. They varied between 2.21 GPa and 3.48 GPa. Z-yarn modulus (E_z-yarn_) might probably be critical values during in vitro, in vivo, or ex vivo experiments due to strong stiffness properties through-the-thickness direction where hydrogel modulus was negligibly small. 

**Figure 13:**
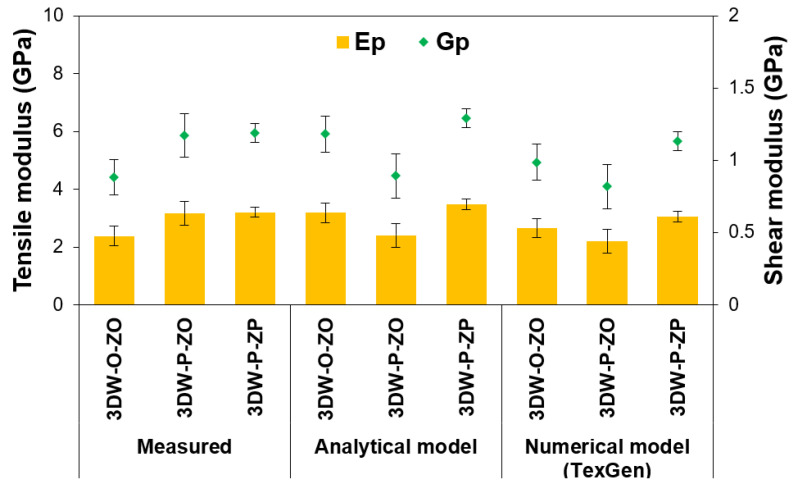
Tensile and shear modulus of all 3D PGA fiber preform scaffold from measured values, analytical model, and FE model (TexGen).

As seen in Table 8 and Figure 13, the shear modulus (G_p_) of 3DW-P-ZP was nearly the highest value and followed by 3DW-P-ZO and 3DW-O-ZO structures. In addition, the warp shear modulus (G_warp-filling_) was the highest compared to the filling (G_filling-z-yarn_) and z-yarn (G_z-yarn-warp_) modulus. The shear moduli obtained from measured, analytic, and numerical models were close to each other. They varied between 0.82 GPa and 1.29 GPa. Z-yarn shear modulus (G_z-yarn-warp_) could probably be critical values during in vitro, in vivo, or ex vivo experiments due to robust stiffness properties through-the-thickness direction where the hydrogel modulus was comparatively small. It was also obtained that the tensile and shear modulus of all 3D PGA fiber preform scaffolds were proportional to their total fiber volume fractions (Figure 13). 

It was found that the tensile strength (σ_ps_) of 3DW-P-ZP was the highest value, followed by 3DW-P-ZO and 3DW-O-ZO structures. In addition, warp strength (*σ*_w_) was the highest compared to the filling (*σ*_f_) and z-yarn (*σ*_z_) strength. The tensile strength obtained from measured, analytic, and numerical models were close to each other. They varied between 27.17 MPa and 42.75 MPa. Z-yarn tensile strength is probably critical values during in vitro, in vivo, or ex vivo experiments due to robust strength properties in the thickness of the scaffolds, which can help provide a microstress environment for cells and influence especially cell adhesion, stimulate cell growth, and proliferation rates of cells for 3D tissue regenerations. On the other hand, the ultimate tensile strength of actual articular cartilage varied from 15–35 MPa [90]. The measured tensile strength of the 3D PGA fiber preform scaffold varied from 29.29–39.49 MPa. The tensile strength values of both actual and 3D PGA fiber scaffolds were near each other. Therefore, it was considered that the tensile strength properties of biodegradable 3D fiber PGA fiber scaffold were approximately matching the native tissue.

### 3.4. Relevance of Possible Results of a Three-Dimensional Preform for the Scaffold of Tissue Engineering

Three-dimensional (3D) biodegradable polyglycolic acid fiber (PGA) preforms were developed for possible applications for the scaffold of 3D tissue regenerations. On the other hand, three-dimensional preform architectures including fiber volume fraction, porosity, stiffness, and strength influenced scaffold properties for tissue engineering. Primarily, 3D tissue regeneration could be possible due to presence of the through-the-thickness continuous polyglycolic acid fiber reinforcement in 3D preform scaffold, which probably stimulates cell contact guidance and mechanical stimulation that match the mechanical properties of regenerated target tissues [9,20,26]. Therefore, various developed 3D polyglycolic acid fiber preforms can be used as a temporary supportive substrate for 3D tissue regeneration, such as articular cartilage, during in vitro, in vivo, or ex-vivo studies. 

### 3.5. Morphology of Preform Structure for Scaffold Results

#### 3.5.1. 3D Plain Woven Preforms

**Figure 14:**
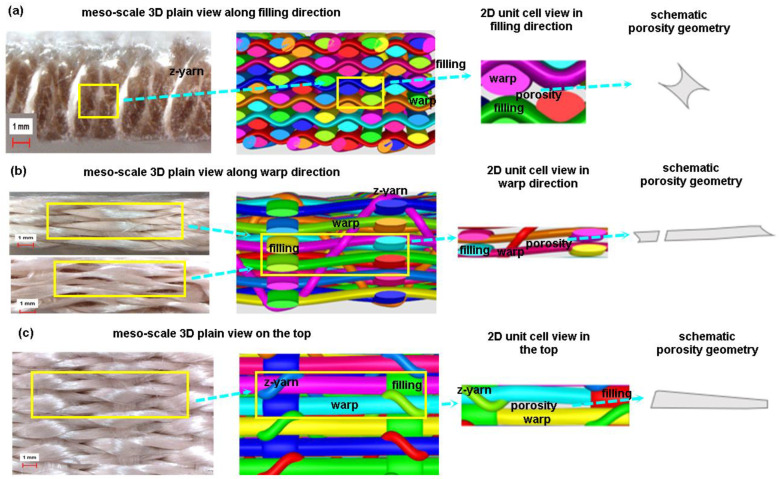
Optical image and schematic view of 3D plain preform architecture (optical microscope image, ×6.7, TexGen). (**a**) Meso-scale 3D preform view along filling direction (optical microscope image, ×6.7, TexGen); (**b**) meso-scale 3D preform view along warp direction (optical microscope image, ×6.7, TexGen); and (**c**) meso-scale 3D preform view on the top (optical microscope image, ×6.7, TexGen).

Actual and schematic unit cells views of 3D plain preform architecture are shown in Figure 14. As exhibited in Figure 14, in the meso-scale 3D preform view along filling (cross-section), polyglycolic acid fiber warp cross-sections were a lenticular shape, and each warp cross-section was almost heterogeneous due to applied inter-fiber pressure during scaffold formation (Figure 14a). However, the porosity shape in the preform based on the virtual TexGen model appeared to have an angular curve-I beam. In the meso-scale 3D preform view along warp (side), polyglycolic acid fiber-filling cross-sections were flat lenticular shape, and each filling cross-section was heterogeneous due to applied warp and z-yarn inter-pressure during scaffold formation (Figure 14b). However, the preform’s porosity shape based on the virtual TexGen model appeared nearly partially rectangular. In the meso-scale 3D preform view on surface (top), polyglycolic acid fiber warp and filling were flat rectangular trajectories, and z-yarns had large arc curvature or angularly deformed arc curvature (criss-cross). They were heterogeneous due to exerted inter-fiber pressure generated during fiber-to-fiber interlacements for scaffold formation (Figure 14c). However, the porosity shape in the preform based on the virtual model featured a nearly trapezoidal beam.

#### 3.5.2. 3D Semi-Interlaced Woven Preforms

**Figure 15:**
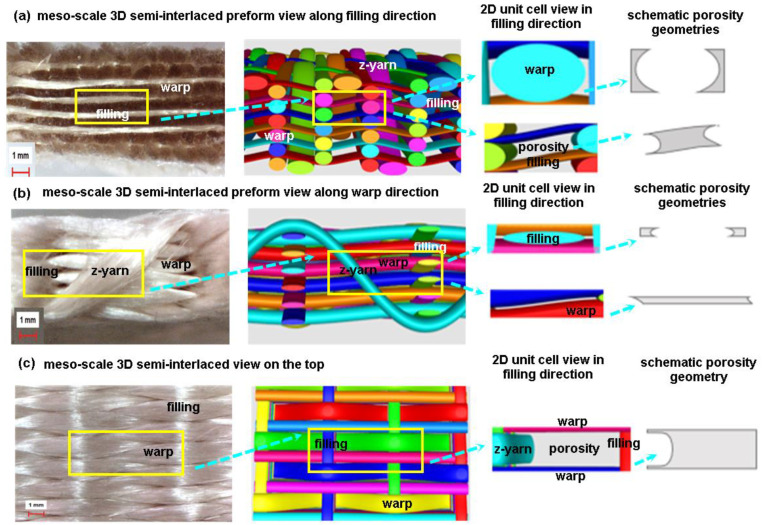
Optical image and schematic view of 3D semi-interlaced preform architecture (optical microscope image, ×6.7, TexGen). (**a**) Meso-scale 3D semi-interlaced preform view along filling direction (optical microscope image, ×6.7, TexGen); (**b**) meso-scale 3D preform view along warp direction (optical microscope image, ×6.7, TexGen); and (**c**) meso-scale 3D preform view on the top (optical microscope image, ×6.7, TexGen).

Actual and schematic unit cells views of 3D semi-interlaced preform architecture are exhibited in Figure 15. As illustrated in Figure 15, in the meso-scale 3D preform view along filling (cross-section), polyglycolic acid fiber warp cross-sections were about ellipse shape, and each warp cross-section was heterogeneous due to applied inter-fiber pressure, which bent yarn cross-sections during scaffold formation (Figure 15a). However, the porosity shape in the preform based on the virtual TexGen model appeared to have concave rectangular beam shapes. In the meso-scale 3D preform view along warp (side), polyglycolic acid fiber filling cross-sections were near to flat lenticular shape and each filling cross-section was heterogeneous due to applied warp and z-yarn inter-pressure causing spreading the PGA filaments around the warp during scaffold formation (Figure 15b). However, the porosity shape in the preform based on the virtual model looked nearly partial trapezoidal or curvy square beams. In the meso-scale 3D preform view on the surface (top), polyglycolic acid fiber warp and filling were flat rectangular trajectories, and z-yarns had large arc curvature. They were heterogeneous due to exerted inter-fiber pressure generated during in-plane fiber-to-fiber interlacements for scaffold formation (Figure 15c). However, the porosity shape in the preform based on the virtual model featured a nearly curvy trapezoidal beam. 

#### 3.5.3. 3D Orthogonal Woven Preforms

**Figure 16:**
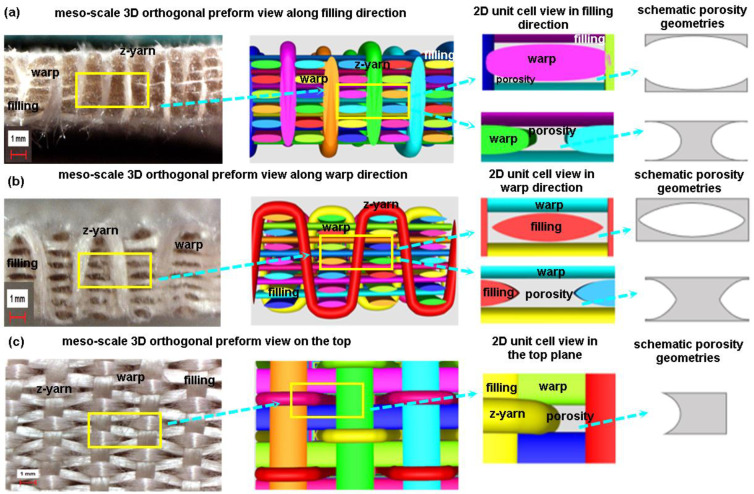
Optical image and schematic view of 3D orthogonal preform architecture (optical microscope image, ×6.7, TexGen). (**a**) Meso-scale 3D orthogonal preform view along filling (optical microscope image, ×6.7, TexGen); (**b**) meso-scale 3D orthogonal preform view along the warp (optical microscope image, ×6.7, TexGen); and (**c**) meso-scale 3D orthogonal preform view on the top (optical microscope image, ×6.7, TexGen).

Actual and schematic unit cells view of 3D orthogonal preform architecture is depicted in Figure 16. As exhibited in Figure 16, in the meso-scale 3D preform view along filling (cross-section), polyglycolic acid fiber warp cross-sections were near to rectangular or square shape, and each warp cross-section was relatively heterogeneous due to applied inter-fiber pressure during scaffold formation (Figure 16a). However, the porosity shape in the preform based on the numerical model (TexGen) appeared to have a concave and angular curve-I beam, which may be considered as a synclastic curvature and revolution of the body. In the meso-scale 3D preform view along warp (side), polyglycolic acid fiber-filling cross-sections were small-size ellipse shape, and each filling cross-section was heterogeneous due to applied warp and z-yarn inter-pressure during scaffold formation (Figure 16b). However, the porosity shape in the preform based on the virtual model appeared nearly an ellipsoid rectangular cylinder. In the meso-scale 3D preform view on the surface (top), polyglycolic acid fiber warp was intra-curvy small scale, whereas filling was large flat rectangular, and z-yarns had sharp arc curvatures. They were heterogeneous due to exerted inter-fiber pressure generated during surface fiber-to-fiber interlacements (Figure 16c). However, the porosity shape in the preform based on the virtual model featured nearly one side curvy rectangular shape.

## 4. Conclusions

Three-dimensional (3D) biodegradable polyglycolic acid (PGA) fiber preforms were made to scaffold three-dimensional tissue regeneration such as articular cartilage. Yarn-to-yarn space, density, and angles of all 3D PGA fiber preform were heterogeneous and demonstrated direction-dependent features (anisotropy). Further, it was obtained that directional crimp ratios were proportional, and the z-yarn crimp ratio (%) was the highest and followed by weft and warp crimp ratios (%). Total volume fractions (*V_fp_*) predicted by the analytic and numerical model of all 3D preform scaffolds exhibited some deviations compared to the measured values because yarn cross-sections in the preform were changed from ideal circular geometry to high-order ellipse (lenticular) due to inter-fiber pressure generated during preform formation. 

The stiffness properties of 3D preform scaffolds varied between 2.21 GPa and 3.48 GPa. Z-yarn modulus (E_z-yarn_) was probably a critical value due to strong stiffness in the through-the-thickness direction where the hydrogel modulus was negligibly small. The tensile strength values of both actual such as articular cartilage and 3D PGA fiber scaffold were near each other. Therefore, it was considered that the tensile strength properties of biodegradable 3D fiber PGA fiber scaffold were approximately matching the native tissue. 

The morphology of the preform scaffold showed that PGA fiber sets were locally distorted and appeared in the scaffold as inconsistent and inhomogeneous continuous fiber forms. Additionally, various porosity shapes in the preform based on the virtual model featured nearly trapezoidal beams, curvy trapezoidal, angular curvy beams, partial or concave rectangular beams, curvy square beams, and ellipsoid rectangular cylinders. Therefore, various developed 3D polyglycolic acid fiber preforms can be used as a temporary supportive substrate for 3D tissue regeneration (articular cartilage) due to through-the-thickness fiber (z-yarn) reinforcement. Future research on 3D preform scaffolds should carry out in vivo, in vitro, or ex vivo environments to test their performance for 3D tissue regeneration.

## Figures and Tables

**Table 1:** Specifications of biodegradable PGA filament and yarn forms.

Fiber Type	Fiber Diameter (μm)	Fiber Density (g/cm^3^)	Tensile Strength (MPa)	Tensile Modulus (GPa)	Elongation at Break (%)	Yarn Linear Density (Tex, Denier)	Yarn Dimension (Width × Thickness) (50 Ends, mm)	Degradation Time (Month)
Polyglycolic acid (PGA, Meta Biomed Inc., Cheongju, Ch‘ungch‘ong-bukto, Republic of Korea)	13.70 [97]	1.50 [97]	60–99.70 [98]	6–7 [98]	1.50–20 [98]	6.22 (56) [97]	1.50 × 0.60	6–12 [97]

**Table 2:** Fiber volume fraction equations for 3D plain woven preform.

Volume Fraction Formulae for 3D Plain Preform		Equation Number	Explanation
$V_{fw}=\frac{V_{w}}{V_{p}}$	$V_{fw}=\frac{\left[NM\left(Td_{f}+\left(T+1\right)d_{z}\right)\left(1+C_{w}\right)\left(\frac{\pi \;d_{w}^{2}}{4}\right)\right]}{\left[\left(Md_{w}+\left(M+1\right)d_{z}\right)\left(Nd_{w}+Nd_{f}\right)\left(Td_{f}+\left(T+1\right)d_{z}\right)\right]}\;$	(8)	Warp volume percent in preform
$V_{ff}\;=\frac{V_{f}}{V_{p}}$	$V_{ff}\;=\frac{\left[TN\left(Md_{w}+Md_{z}\right)\left(1+C_{f}\right)\left(\frac{\pi \;d_{f}^{2}}{4}\right)\right]\;}{\left[\left(Md_{w}+\left(M+1\right)d_{z}\right)\left(Nd_{w}+Nd_{f}\right)\left(Td_{f}+\left(T+1\right)d_{z}\right)\right]}$	(9)	Filling volume percent in preform
$V_{fz}\;=\frac{V_{z}}{V_{p}}$	$V_{fz}\;=\frac{\left[TM\left(Nd_{w}+Nd_{f}+1d_{z}\right)(1+C_{z})\left(\frac{\pi \;d_{z}^{2}}{4}\right)\right]}{\left[\left(Md_{w}+\left(M+1\right)d_{z}\right)\left(Nd_{w}+Nd_{f}\right)\left(Td_{f}+\left(T+1\right)d_{z}\right)\right]}\;$	(10)	Z-fiber volume percent in preform
$V_{fp}=V_{fw}+V_{ff}+V_{fz}\;$	$V_{fp}=\frac{V_{w}+V_{f}+V_{z}}{V_{p}}\;\;$	(11)	Preform volume percent
$V_{tpr}=1-V_{fp}$	$V_{tpr}=1-V_{fp}$	(12)	Porosity percent in preform

Where *V_p_* is the unit cell volume; *N* is the total number of warp yarns per row; *M* is the total number of warp yarns per column; *T* is the weft density number in the unit cell; *d_w_* is the warp yarn diameter; *d_f_* is the weft yarn diameter; *d_z_* is the z-yarn diameter; *C_w_* is warp yarn crimp; *C_f_* is weft yarn crimp; *C_z_* is z-yarn crimp; *V_w_* is the warp yarn volume in the unit cell; *V_f_* is the weft yarn volume in the unit cell; *V_z_* is the z-yarn volume in the unit cell; *V_fw_* is the warp yarn volume ratio; *V_ff_* is the weft yarn volume ratio; V_fz_ is the z-yarn volume ratio and *V_fp_* is the total volume ratio and *V_tpr_* is the porosity volume ratio.

**Table 3:** Fiber volume fraction equations for 3D semi-interlaced woven preform.

Volume Fraction Formulae for 3D Semi-Interlaced Preform		Equation Number	Explanation
$V_{fw}=\frac{V_{w}}{V_{p}}$	$V_{fw}=\frac{\left[NM\left(Td_{f}+\left(T+1\right)d_{z}\right)\left(1+C_{w}\right)\left(\frac{\pi \;d_{w}^{2}}{4}\right)\right]}{\left[\left(Md_{w}+\left(M+1\right)d_{z}\right)\left(Nd_{w}+Nd_{f}\right)\left(Td_{f}+\left(T+1\right)d_{z}\right)\right]\;}$	(13)	Warp volume percent in preform
$V_{ff}\;=\frac{V_{f}}{V_{p}}$	$V_{ff}\;=\frac{\left[TN\left(Md_{w}+Md_{z}\right)\left(1+C_{f}\right)\left(\frac{\pi \;d_{f}^{2}}{4}\right)\right]}{\left[\left(Md_{w}+\left(M+1\right)d_{z}\right)\left(Nd_{w}+Nd_{f}\right)\left(Td_{f}+\left(T+1\right)d_{z}\right)\right]\;}\;$	(14)	Filling volume percent in preform
$V_{fz}\;=\frac{V_{z}}{V_{p}}$	$V_{fz}\;=\frac{\left[TM\left(Nd_{w}+Nd_{f}+1d_{z}\right)\left(\frac{\pi \;d_{z}^{2}}{4}\right)\right]}{\left[\left(Md_{w}+\left(M+1\right)d_{z}\right)\left(Nd_{w}+Nd_{f}\right)\left(Td_{f}+\left(T+1\right)d_{z}\right)\right]\;}\;$	(15)	Z-fiber volume percent in preform

**Table 4:** Fiber volume fraction equations for 3D orthogonal woven preform.

Volume Fraction Formulae for 3D Orthogonal Preform		Equation Number	Explanation
$V_{fw}=\frac{V_{w}}{V_{p}}$	$V_{fw}=\frac{\left[\left(NMTd_{f}+(T+1)d_{z}\right)\left(\frac{\pi \;d_{w}^{2}}{4}\right)\right]}{\left[\left(Md_{w}+(M+1)d_{z}\right)\left(Nd_{w}+(N+1)d_{f}\right)\left(Td_{f}+(T+1)d_{z}\right)\right]}\;$	(16)	Warp volume percent in preform
$V_{ff}\;=\frac{V_{f}}{V_{p}}$	$V_{ff}=\frac{\left[\left(T\left(N+1\right)\left(Md_{w}+Md_{z}\right)\right)\;\left(\frac{\pi \;d_{f}^{2}}{4}\right)\right]}{\left[\left(Md_{w}+(M+1)d_{z})(Nd_{w}+(N+1)d_{f})(Td_{f}+(T+1)d_{z}\right)\right]}\;$	(17)	Filling volume percent in preform
$V_{fz}\;=\frac{V_{z}}{V_{p}}$	$V_{fz}=\frac{\left[\left(TM\left(Nd_{w}+\left(N+1\right)d_{f}+d_{z}\right)\right)\left(\frac{\pi \;d_{z}^{2}}{4}\right)\right]}{\left[\left(Md_{w}+(M+1)d_{z})(Nd_{w}+(N+1)d_{f})(Td_{f}+(T+1)d_{z}\right)\right]}\;$	(18)	Z-fiber volume percent in preform

**Table 5:** Yarn-to-yarn space, density, and angles of all 3D PGA fiber woven preform structures.

Yarn-to-Yarn Space											
Preform Structure	Layer Number	Total Yarn Number			Structure		Yarn–Yarn Distance (Top × Side × Cross-Section)				
		Warp (N × M)	Filling (N + 1, N)	Z-Yarn (M + 1)	Width × Thickness (S_w_ × S_t_, mm)		Warp-Warp (mm)	Filling-Filling (mm)		Z-Yarn-Z-Yarn (mm)	
3DW-O-ZO	6	6 × 20	7 × 1	1 × 21	28.00 × 5.66		1 × 0.5 × 1	3 × 7 × 0.3		1 × 6 × 1	
3DW-P-ZO		6 × 20	6 × 1	1 × 21	20.30 × 3.99		1 × 1 × 1	8 × 8 × 1		1 × 10 × 1	
3DW-P-ZP		6 × 20	6 × 1	1 × 20	27.66 × 4.00		1 × 0.8 × 1	5 × 5 × 0.3		1 × 11 × 1	
**Density**											
**Preform Structure**	**Layer Number**	**Total Yarn Number**			**Structure**		**Preform Density** **(Top × Side × Cross-Section)**			**Preform Areal Density** **(g/cm^3^)**	
		**Warp** **(N × M)**	**Filling** **(N + 1, N)**	**z-yarn** **(M + 1)**	**Length × Width × Thickness** **(S_l_ × S_w_ × S_t_, mm)**		**Warp** **(ends/10 mm)**	**Filling** **(ends/10 mm)**	**Z-yarn** **(ends/10 mm)**		
3DW-O-ZO	6	6 × 20	7 × 1	1 × 21	100 × 28.00 × 5.66		8 × – × 8	4 × 4 × –	6 × 2 × 6	0.55 ± 0.10	
3DW-P-ZO		6 × 20	6 × 1	1 × 21	125 × 20.30 × 3.99		9 × – × 9	2 × 2 × –	9 × 1 × 9	0.73 ± 0.10	
3DW-P-ZP		6 × 20	6 × 1	1 × 20	100 × 27.66 × 4.00		9 × – × –	2 × 2 × –	8 × 1 × 8	0.74 ± 0.10	
**Angles**											
**Preform Structure**	**Layer Number**	**Total Yarn Number**			**Warp Angle**		**Filling Angle**		**Z-Yarn Angle**		
		**Warp**	**Filling**	**z-yarn**	**(θ_wc_°)–(θ_wm_°)**	**(θ_ws_°)**	**(θ_fc_°)**	**(θ_fm_°)–(θ_fs_°)**	**(θ_zc_°)**	**(θ_zm_°)**	**(θ_zs_°)**
3DW-O-ZO	6	6 × 20	7 × 1	1 × 21	0–0	29 ± 14.50	90	0–90	70	90 ± 5.77	0
3DW-P-ZO		6 × 20	6 × 1	1 × 21	0–0	0	90	0–90	40	90 ± 5.77	0
3DW-P-ZP		6 × 20	6 × 1	1 × 20	* −75	15 ± 14.50	13	0–90	40	80 ± 5.77	10 ± 5.77

* It could not measure.

**Table 6:** Crimp and fiber length measurements of all 3D PGA fiber woven preforms.

Crimp										
Preform Structure	Layer Number	Uncrimp Yarn Length			Structure			Crimp		
		Warp (l_w_, mm)	Filling (l_f_, mm)	Z-Yarn (l_zt_, mm)	Length (S_l_, mm)	Width (S_w_, mm)	Thickness (S_t_, mm)	Warp (C_w_, %)	Filling (C_f_, %)	Z-Yarn (C_z_, %)
3DW-O-ZO	6	51	30	7	50	28	6.36	2.00 ± 1.75	7.14 ± 3.57	10.06 ± 20.06
3DW-P-ZO		30	20	8	29	18	6.00	3.44 ± 1.75	11.11 ± 3.57	33.33 ± 20.06
3DW-P-ZP		38	24	15	36	23	10.00	5.55 ± 1.75	14.28 ± 3.57	50.00 ± 20.06
**Yarn Length**										
**Preform Structure**	**Layer Number**	**Total Yarn Number**			**Uncrimp Warp Length** **(l_w_,mm)**	**Uncrimp Filling Length** **(l_f_, mm)**	**Uncrimp Z-Yarn Length**			
		**Warp**	**Filling**	**Z-Yarn**			**Z-Yarn Arc Length** **(l_za_, mm)**		**Z-Yarn Length** **(l_z_, mm)**	**Total Z-Yarn Length** **(l_zt,_ mm)**
3DW-O-ZO	6	6 × 20	7 × 1	1 × 21	151	46.34	6		6.36	12.36
3DW-P-ZO		6 × 20	6 × 1	1 × 21	140	40.30	10		3.99	13.99
3DW-P-ZP		6 × 20	6 × 1	1 × 20	114	47.66	5		12.50	17.50

**Table 7:** Fiber volume fractions based on measured values, analytic modeling, and FE model (TexGen) of all 3D PGA fiber woven preforms.

Fiber Volume Fraction (%)	3D PGA Fiber Woven Preform Structures		
	3DW-O-ZO	3DW-P-ZO	3DW-P-ZP
**Measured values**			
Warp directional fiber volume fraction (*V_fw_*)	20.06 ± 4.15	30.24 ± 4.15	24.87 ± 4.15
Weft directional fiber volume fraction (*V_ff_*)	7.03 ± 2.93	9.71 ± 2.93	14.15 ± 2.93
Z-directional fiber volume fraction (*V_fz_*)	9.55 ± 0.83	8.03 ± 0.83	9.96 ± 0.83
Warp directional porosity (*V_wpr_*)	21.11 ± 2.37	17.11 ± 2.37	16.89 ± 2.37
Weft directional porosity (*V_fpr_*)	21.11 ± 2.37	17.11 ± 2.37	16.89 ± 2.37
Z-directional porosity (*V_zpr_*)	21.11 ± 2.37	17.11 ± 2.37	16.89 ± 2.37
Total preform scaffold fiber volume fraction (*V_fp_*)	36.66 ± 7.12	48.66 ± 7.12	49.33 ± 7.12
Total preform scaffold porosity fraction (*V_tpr_*)	63.34 ± 7.12	51.34 ± 7.12	50.67 ± 7.12
**Analytical model**			
Warp directional fiber volume fraction (*V_fw_*)	17.67 ± 1.32	19.72 ± 1.32	17.23 ± 1.32
Weft directional fiber volume fraction (*V_ff_*)	13.09 ± 0.93	14.17 ± 0.93	14.96 ± 0.93
Z-directional fiber volume fraction (*V_fz_*)	18.32 ± 9.72	3.19 ± 9.72	21.33 ± 9.72
Warp directional porosity (*V_wpr_*)	16.97 ± 2.83	20.97 ± 2.83	15.49 ± 2.83
Weft directional porosity (*V_fpr_*)	16.97 ± 2.83	20.97 ± 2.83	15.49 ± 2.83
Z-directional porosity (*V_zpr_*)	16.97 ± 2.83	20.97 ± 2.83	15.49 ± 2.83
Total preform scaffold fiber volume fraction (*V_fp_*)	49.08 ± 8.50	37.08 ± 8.50	53.52 ± 8.50
Total preform scaffold porosity (*V_tpr_*)	50.92 ± 8.50	62.92 ± 8.50	46.48 ± 8.50
**TexGen model**			
Warp directional fiber volume fraction (*V_fw_*)	20.00 ± 5.85	22.00 ± 5.85	31.00 ± 5.85
Weft directional fiber volume fraction (*V_ff_*)	14.00 ± 4.60	5.00 ± 4.60	11.20 ± 4.60
Z-directional fiber volume fraction (*V_fz_*)	7.00 ± 1.15	7.00 ± 1.15	5.00 ± 1.15
Warp directional porosity (*V_wpr_*)	19.66 ± 2.51	22.66 ± 2.51	17.66 ± 2.51
Weft directional porosity (*V_fpr_*)	19.66 ± 2.51	22.66 ± 2.51	17.66 ± 2.51
Z-directional porosity (*V_zpr_*)	19.66 ± 2.51	22.66 ± 2.51	17.66 ± 2.51
Total preform scaffold fiber volume fraction (*V_fp_*)	41.00 ± 6.50	34.00 ± 6.50	47.00 ± 6.50
Total preform scaffold porosity (*V_tpr_*)	59.00 ± 6.50	68.00 ± 6.50	53.00 ± 6.50

**Table 8:** Stiffness and strengths of 3D PGA fiber preform scaffolds based on measured values, analytical model, and numerical models (TexGen).

Fiber Volume Fractions	Preform Structure	Tensile Modulus (GPa)				Shear Modulus (GPa)			
		E_warp_ (E_11_)	E_filling_ (E_22_)	E_z-yarn_ (E_33_)	E_p_	G_warp-filling_ (G_12_)	G_filling-z-yarn_ (G_23_)	G_z-yarn-warp_ (G_13_)	G_p_
Measured	3DW-O-ZO	1.30 ± 0.27	0.46 ± 0.19	0.62 ± 0.05	2.38 ± 0.34	0.48	0.17	0.23	0.88 ± 0.12
	3DW-P-ZO	1.97 ± 0.27	0.63 ± 0.19	0.52 ± 0.05	3.16 ± 0.41	0.73	0.23	0.19	1.17 ± 0.15
	3DW-P-ZP	1.62 ± 0.27	0.92 ± 0.19	0.65 ± 0.05	3.21 ± 0.18	0.60	0.34	0.24	1.19 ± 0.07
Analytical model	3DW-O-ZO	1.15 ± 0.07	0.85 ± 0.05	1.19 ± 0.52	3.19 ± 0.34	0.43	0.32	0.44	1.18 ± 0.12
	3DW-P-ZO	1.28 ± 0.07	0.92 ± 0.05	0.21 ± 0.52	2.41 ± 0.41	0.48	0.34	0.10	0.89 ± 0.15
	3DW-P-ZP	1.12 ± 0.07	0.97 ± 0.05	1.39 ± 0.52	3.48 ± 0.18	0.42	0.36	0.51	1.29 ± 0.07
FE model (TexGen)	3DW-O-ZO	1.30 ± 0.31	0.91 ± 0.24	0.46 ± 0.06	2.66 ± 0.34	0.48	0.34	0.17	0.99 ± 0.12
	3DW-P-ZO	1.43 ± 0.31	0.33 ± 0.24	0.46 ± 0.06	2.21 ± 0.41	0.53	0.12	0.17	0.82 ± 0.15
	3DW-P-ZP	2.02 ± 0.31	0.73 ± 0.24	0.33 ± 0.06	3.06 ± 0.18	0.75	0.27	0.12	1.13 ± 0.07
**Fiber Volume Fractions**	**Preform Structure**	**Poisson Ratios**				**Tensile Strengths** **(MPa)**			
		**ν_warp-filling_** **ν_12_**	**ν_filling-z-yarn_** **ν_23_**	**ν_z-yarn-warp_** **ν_13_**	**ν_p_**	**σ_warp_** **σ_11_**	**σ_filling_** **σ_22_**	**σ_z-yarn_** **σ_33_**	**σ_ps_**
Measured	3DW-O-ZO	0.44	0.45	0.45	0.42	16.04 ± 3.32	5.64 ± 2.34	7.65 ± 0.66	29.29 ± 4.11
	3DW-P-ZO	0.43	0.45	0.45	0.41	24.16 ± 3.32	7.78 ± 2.34	6.44 ± 0.66	38.87 ± 5.04
	3DW-P-ZP	0.43	0.44	0.45	0.40	19.88 ± 3.32	11.32 ± 2.34	7.98 ± 0.66	39.40 ± 2.15
Analytical model	3DW-O-ZO	0.44	0.44	0.44	0.40	14.13 ± 0.87	10.47 ± 0.61	14.65 ± 6.34	39.20 ± 4.11
	3DW-P-ZO	0.44	0.44	0.45	0.42	15.77 ± 0.87	11.34 ± 0.61	2.57 ± 6.34	29.62 ± 5.04
	3DW-P-ZP	0.44	0.44	0.43	0.40	13.78 ± 0.87	11.97 ± 0.61	17.05 ± 6.34	42.75 ± 2.15
FE model (TexGen)	3DW-O-ZO	0.44	0.44	0.45	0.41	15.99 ± 3.82	11.20 ± 3.00	5.61 ± 0.75	32.75 ± 4.11
	3DW-P-ZO	0.43	0.45	0.45	0.42	17.59 ± 3.82	4.02 ± 3.00	5.61 ± 0.75	27.17 ± 5.04
	3DW-P-ZP	0.42	0.45	0.45	0.41	24.77 ± 3.82	8.97 ± 3.00	4.02 ± 0.75	37.54 ± 2.15
**Articular cartilage**									
**Tensile modulus** **(MPa, 10% *ε*)**			**Complex shear modulus** **(MPa)**			**Ultimate tensile strengths** **(MPa)**			
5.00–25.50 [90]			0.20–2.00 [90]			15–35 [90]			

## Data Availability

Data is provided in the article.
